# Reply: Calcified vs Noncalcified Plaque in HIV Revisited: Insights From REPRIEVE

**DOI:** 10.1016/j.jacadv.2025.102052

**Published:** 2025-08-08

**Authors:** Michael T. Lu, Heather J. Ribaudo, Pamela S. Douglas, Steven K. Grinspoon

**Affiliations:** a Cardiovascular Imaging Research Center, Department of Radiology, Massachusetts General Hospital and Harvard Medical School, Boston, Massachusetts, USA; b Center for Biostatistics in AIDS Research, Harvard T.H. Chan School of Public Health, Boston, Massachusetts, USA; c Duke Clinical Research Institute, Duke University School of Medicine, Durham, North Carolina, USA; d Massachusetts General Hospital and Harvard Medical School, Boston, Massachusetts, USA

We thank Dr Razvi and colleagues for their comments on our paper.^1^ Noncalcified plaque (NCP) was the prespecified primary outcome of the REPRIEVE (The Randomized Trial to Prevent Vascular Events in HIV) Mechanistic Substudy. NCP volume was chosen as the primary outcome because it is the biologically active component of coronary plaque with the potential to decrease with statin therapy. In contrast, statins are known to increase the coronary artery calcium score (CACS). Some people will have substantial NCP even with a CACS of zero, particularly in younger persons, women, and in persons with HIV (PWH).^2^

**Figure 1:**
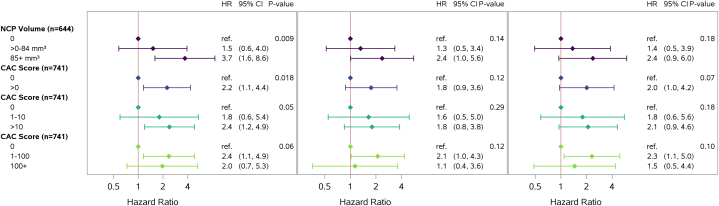
**Estimated Baseline Plaque Effect on Subdistribution Hazard of MACE** (Left) Unadjusted. (Middle) Adjusted for ASCVD risk score. (Right) Adjusted for individual ASCVD risk score components. Subdistribution HR estimates with 95% CIs. For visual purposes, the x-axis is shown in the log scale. ASCVD = atherosclerotic cardiovascular disease; CAC = coronary artery calcium; MACE = major adverse cardiovascular events; NCP = noncalcified coronary plaque.

In response to Dr Razvi and colleagues, we now provide additional cut points for the CACS in Figure 1. These data suggest that while the hazard of MACE is higher with increasing cutoffs for NCP volume 0, >0 to 84, and >84 mm^3^, the same trend is not apparent with CAC score cut points (>0, >10, 1-100), regardless of CAC cutoff used. However, wide CIs for the associations prevent definitive conclusions comparing assessment techniques with respect to MACE.

The parent REPRIEVE trial provided strong evidence that pitavastatin yields a 36% reduction in MACE over a median of 5.6 years.^3^^,^^4^ In 2024, the DHHS ARV (Department of Health and Human Services Antiretroviral) guidelines panel issued a strong recommendation for at least moderate intensity statin for primary prevention in PWH aged 40 to 75 years with a 10-year atherosclerotic cardiovascular disease risk ≥5%. For atherosclerotic cardiovascular disease risk <5%, the guideline gives a lower-grade recommendation favoring initiating statin, taking into account other factors that may increase risk.^5^

Imaging with coronary CTA or CACS is not yet recommended for asymptomatic PWH who fall under these guidelines. REPRIEVE demonstrated that statins prevent MACE in HIV, without any imaging to select participants. Our data advance the field, showing that NCP related most strongly to MACE and was observed in approximately 20% of PWH with a calcium score of 0. Whether coronary imaging should be employed in general, optimal timing, groups for whom imaging is most useful, and whether the additional cost, complexity, and radiation dose of coronary CTA over CACS^2^ is warranted among PWH will require further study.

A comparison to the recently developed PREVENT (Predicting Risk of cardiovascular disease EVENTs) score in REPRIEVE is the subject of another analysis.
